# Facial Skincare Routine Adherence in the General Population

**DOI:** 10.7759/cureus.75810

**Published:** 2024-12-16

**Authors:** Leah Cliatt, Joanna Petrides

**Affiliations:** 1 Dermatology, Rowan-Virtua School of Osteopathic Medicine, Stratford, USA; 2 Family Medicine, Rowan-Virtua School of Osteopathic Medicine, Stratford, USA

**Keywords:** beauty, dermatologic agents, dermatology, face cleansing, face washing, female, hygiene, skin

## Abstract

Introduction

The COVID-19 pandemic sparked an interest in skincare with the closure of spas and salons. Skincare, one of TikTok's most popular dermatology-related hashtags, received hundreds of millions of views. The American Academy of Dermatology (AAD) shared facial cleansing recommendations; however,  how many people follow them is unclear. Studies have shown a good daily facial cleansing and moisturizing routine can increase microbiome diversity and skin hydration. This can be beneficial in conditions like psoriasis, eczema, and acne.

Purpose

The aim of the study is to assess how well people follow the AAD recommendations and evaluate any differences in this behavior by gender.

Methods

A 19-question survey was designed and administered utilizing Qualtrics. The questions included demographic information, facial cleansing practices, and motivation for skincare routine. The survey was distributed via Rowan Email and on various social media platforms (GroupMe, Instagram, etc.) to target the general population. The data was analyzed using SPSS.

Results

One hundred twenty-four responses were collected from 91 female-identifying and 33 male-identifying participants. There were statistically significant differences between genders for the use of non-alcoholic gentle cleanser (p<0.001), use of moisturizer after washing the face (p<0.001), washing the face after sweating (p<0.001), and using warm water (p=0.026). No statistically significant difference was seen for face washing occurrence between genders (p=0.098). Statistically significant differences were seen between genders for motivation: hygiene (p<0.001), beauty/anti-aging (p<0.001), and health (p=0.004).

Conclusion

Individuals who identify as female may be more likely to adhere to AAD facial skincare recommendations. This could be a result of self-reported motivations such as hygiene, beauty, and health.

## Introduction

The COVID-19 pandemic greatly impacted the world in many ways, from how we perceive health to the way we interact socially. One of the unexpected changes was an increased interest in skincare routines due to the closure of spas and increased usage of masks. Many people turned to social media during the pandemic for tips on facial skin care [1]. A survey conducted in Poland with 412 participants between the ages of 18-29 years found that 58% of respondents claimed they now focus more on their facial skincare compared to pre-pandemic. This study also found that 48% of women looked to social media for facial skincare tips [1]. Another post-pandemic survey study had comparable results and found that while people focused more on facial skincare during the pandemic, most participants did not consult a dermatologist about their regimen changes [2]. The American Academy of Dermatology (AAD) has recommendations on its website; however, it is unclear how many people follow them. According to the AAD, a routine is fully beneficial if it is done properly. The AAD recommends using a gentle cleanser and moisturizer twice daily (morning and evening) for the best results. This is because there are many known benefits of facial cleansing and daily moisturizing.

Humans have diverse commensal microbes on facial skin that are integral in preventing harmful pathogens from colonizing the skin [3]. Patients with common skin conditions such as psoriasis, eczema, acne, and atopic dermatitis often have an unbalanced microbiome [4]. A study placed 25 women on a facial cleanser and moisturizer compound routine used twice daily (morning and evening) for 4 weeks. At the end of the study, the participants had a higher alpha diversity of their facial microbiome, indicating more microbial diversity and healthy skin [5]. Another important factor in skin care is the hydration of the stratum corneum. Adequate hydration leads to reduced redness, cracking, and wrinkles [3]. Furthermore, an increase in hydration levels leads to balanced pH and sebum levels. These two studies indicate a correlation between hydration levels and microbiome diversity. 

During the COVID-19 pandemic, there was increased usage of face masks. A randomized control trial was done on 21 female participants who wore masks for at least six hours daily for one week. Half of their mask-covered face was treated with a moisturizer, and the other half was not. The results showed that areas covered by the mask but without the daily usage of moisturizer led to the accumulation of humidity underneath the skin and a weakened stratum corneum. However, on the side with daily moisturizer use, there was decreased trans-epidermal water loss [6]. Further evidence of using a cleanser and moisturizer was seen in another randomized control trial, which included 52 participants with moderate to severe dry skin. Participants were either placed in a group with gentle cleanser use or gentle cleanser with moisturizer for two weeks. Participants who used a gentle cleanser with moisturizer showed improvement in erythema, scale, and fissures. In addition, they reported improved skin texture and a decrease in dryness/pruritus [7]. These studies support the clear benefit of following AAD’s recommendations for face washing and moisturizing. 

While it is evident that facial cleansing is important for hygiene, it is unknown how many people properly follow the AAD recommendations of facial cleansing and moisturizing. COVID-19 prompted an increase in the usage of social media for skincare routines; however, it is unknown how many of these sources are reputable. This has prompted the AAD, dermatologists, and dermatology residency programs to use social media to educate and guide the public [8]. As a result of this shift in information availability, this current project aimed to gain an increased understanding of the general population’s practices of proper facial cleansing, where they receive their facial hygiene information, and what motivates them to have a skincare routine (if they have one). Gaining an understanding of these factors can help guide healthcare providers in having conversations with patients in the future regarding the importance of having a proper skincare routine and sources for this information. 

We hypothesized that the general population does not follow the facial cleansing recommendations by the AAD and does not have a consistent daily face cleansing routine. We also expect females to have a more consistent face-cleansing routine than males. 

This article was previously accepted as an abstract and presented as a poster at the Atlantic Dermatology Conference on April 19-21, 2024 and at Rowan-Virtua School of Osteopathic Medicine Research Day on May 2, 2024, respectively. 

## Materials and methods

The target of this project was the general population and Rowan-Virtua School of Osteopathic Medicine Research (SOM) students (who were considered part of the general population). The subjects were limited to those between the ages of 18-89. The study was designed and administered using Qualtrics. Participants used a link or QR code provided to them on a flyer or social media post to access the anonymous survey. Social media posts were made on GroupMe, Reddit, Instagram, and Facebook, and through Rowan-Virtua SOM email distribution. The survey was completed on each participant’s personal device. The participants were recruited voluntarily and not compensated for participating in the study. Rowan-Virtua School of Osteopathic Medicine Institutional Review Board issued the approval for the study with the approval number PRO-2023-246.

A total of 136 responses were recorded between July 2023 and November 2023. Participants who did not complete the survey were excluded from the study analysis. 124 participants completed a 19-question survey that assessed demographics and facial cleansing and moisturizing routines (Appendix A). Participants were presented with an alternate consent form prior to accessing the survey; only those who indicated consent to participate were permitted to move on to the survey. The next five questions were demographic and socioeconomic questions (age, gender, race, highest level of education, and marital status). The next two questions asked the participants what their skin type is and if they have a skincare routine. Seven questions were developed using their website's AAD recommendations for facial cleansing. The facial cleansing recommendations on AAD’s website, which were used to formulate the questions, are shown in Table 1. Finally, four questions focused on motivation for skincare hygiene, COVID-19's impact on skincare, where participants receive their skincare hygiene information, and additional skincare products used. The data was then analyzed using Qualtrics, and data was exported to Excel for further analysis in SPSS [9]. The seven questions to assess face-washing practices in the general population were designed based on the AAD Face Washing recommendations on their website [10]. 

**Table 1:** Face washing recommendations on AAD's website.

AAD Face Washing Recommendations	
Use gentle a non-alcoholic cleanser	Apply a moisturizer after washing face
Use fingertips to apply cleanser	Wash face twice a day (morning/night)
Use warm water to wash face	Wash face after sweating

## Results

A total of 136 responses were recorded between July 2023 and November 2023. Participants who did not complete the survey were excluded from the study analysis. Additionally, three participants who answered the question, “What gender do you identify as?” as non-binary preferred not to answer, were excluded from the study due to insufficient responses for each respective category to be included in the analysis. After excluding the outliers listed above, a total of 124 responses were included in the analysis. Of the 124 responses, 91 were female-identifying participants, and 33 were male-identifying participants. There were 77 respondents between the ages of 18-29, 31 respondents between the ages of 30-49, 14 between the ages of 50-69, and 2 above the age of 70. The data was analyzed using Chi-squared analysis on SPSS. 

**Figure 1:**
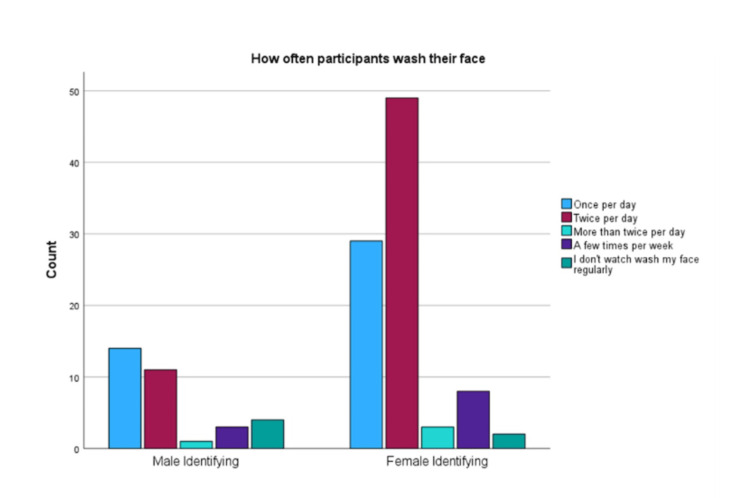
Self-reported occurrence of face-washing. Graph showing how often participants wash their face in a day based on gender. This was a multiple-choice question. There was no statistically significant difference between female-identifying and male-identifying participants (p=0.098). A chi-squared analysis was performed to determine significance. X^2 ^(4, N=124)=7.821, p=0.098, *p<0.05 was considered significant.

For questions on facial cleansing based on AAD recommendations, there was a statistically significant difference between genders for using a gentle non-alcoholic cleanser (p<0.001), using fingertips to apply a cleanser (p<0.001), applying a moisturizer after washing face (p<0.001), washing face after sweating (p<0.001) and using warm water to wash face (p=0.026). As Figure 1 shows, when asked how often participants wash their faces, there was no significant difference between genders (p=0.098). Additionally, no significant difference between ethnicities or education levels in following AAD recommendations for face washing was observed. 

**Figure 2:**
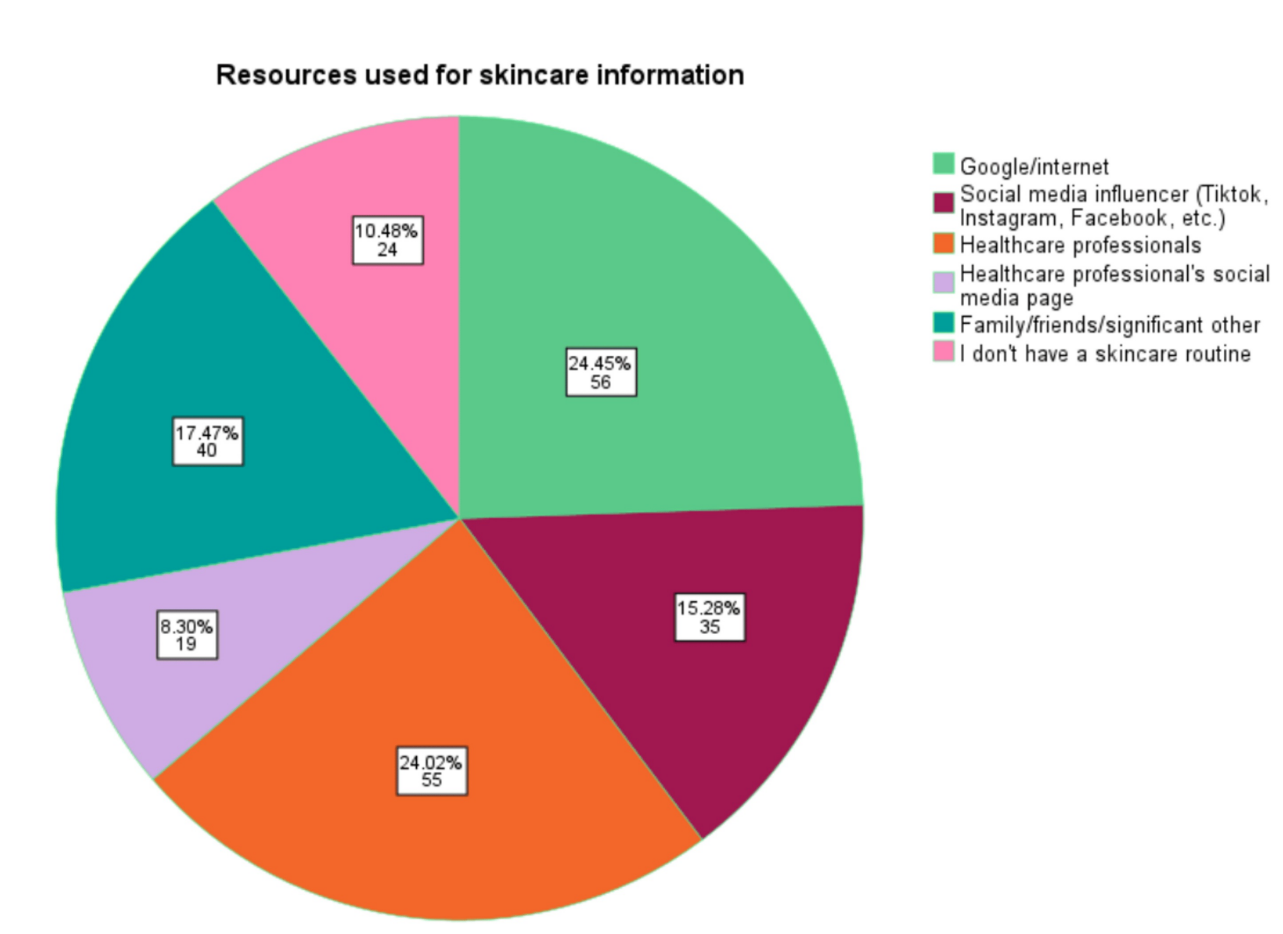
Resources used for skincare information. Pie chart showing the different resources used to receive skincare routine information by participants. Participants were instructed to select all that apply for this question. Percentage of participants and N are reported in each slice. Of the 124 participants, 24.02% of participants selected healthcare professional and 8.30% selected healthcare professional's social media as their resource for receiving skincare information. A chi-squared analysis was performed to determine gender significance in choosing healthcare professionals or healthcare professional's social media page. X^2 ^(1, N=124)=13.987, p<0.001 for healthcare professionals. X^2^ (1, N=124)=5.237, p< 0.022 for healthcare professional's social media, p-value considered significant at p<0.05.

There was a statistically significant difference between female-identifying and male-identifying respondents receiving their skincare routine information from a healthcare professional (p<0.001). Females relied more on healthcare professionals for their skincare information than males. Figure 2 shows the resources that participants use to receive information on their skincare routine. 

As shown in Table 2, for the question asking, “What motivates you to have a skincare routine,” statistically significant results were seen for female skincare routine motivation: hygiene (p<0.001), beauty/anti-aging (p<0.001), and health (p=0.004). 

**Table 2:** Items with statistical difference between male/female gender. Table showing difference in routine and motivation between male and female gender. Table includes p-value, chi-squared value, and degree of freedom (df). p-value<0.05 was considered significant.

Differences in routine	p-value	Chi-squared value	df	Differences in motivation	p-value	Chi-squared value	df
Using a gentle, non-alcoholic cleanser	p<0.001	28.039	3	Hygiene	p< 0.001	15.568	1
Applying moisturizer after washing the face	p< 0.001	19.856	1	Beauty/Anti-aging	p<0.001	12.507	1
Washing face after sweating	p< 0.001	15.157	1	Health	p=0.001	8.368	1
Using warm water	p= 0.026	9.235	3	Confidence	p=0.073	3.209	1
Face washing occurrence	p= 0.098	7.821	4	Family/significant other	p=0.202	1.625	1

## Discussion

Female-identifying participants followed AAD recommendations for face washing more closely than male-identifying participants. Several factors could contribute to this finding. One reason could be that female-identifying participants are more likely to receive their information from healthcare professionals (Figure 2). Additionally, female-identifying participants are motivated by beauty/anti-aging, hygiene, and health. It was unclear what factors motivate men to have a skincare routine. 

Proper face washing is important for healthy skin. It has been shown to improve the skin microbiome and reduce the severity of many different skin conditions, such as acne, psoriasis, and eczema [4]. A patient’s skin type (normal, dry, oily, or combination) determines what kind of cleanser should be utilized. For oily skin, a gel-based cleanser is preferred, and for dry or normal skin, a lotion-based cleanser is preferred [11]. Most of the participants in our study were aware of their skin type, however it is unclear if they were aware what type of cleanser is the best for their skin type. Future research can inquire about this factor when updating the survey. 

Studies have shown that males can equally benefit from a face-washing routine. A clinical study on male and female subjects with acne vulgaris between the ages of 12-35 showed the benefits of utilizing a cleanser and having a skincare routine. In this study, subjects used a cleanser, toner, and acne treatment twice daily in the morning and evening over a six-week period. Results showed significant improvement in acne in both genders [12]. Additionally, skin sebum has been shown to be higher in males than females [13]. Another study with 29 male subjects showed that routine twice-daily shave cleanser, post-shave treatment, and day protection for 4 weeks improved pore lines, razor burn, and folliculitis. Moreover, the moisture content of the stratum corneum had improved as well. [14]. Therefore, it is important that males have a robust face-cleansing routine as well. The current body of research, along with this current project, demonstrates the need for males to be educated on and informed about the benefits of a daily face-washing routine. 

In our study, it was unclear what factors motivate males to have a skincare routine. In order to help males adopt a consistent skincare routine, it would be beneficial to understand what factors would motivate them. Efforts should be made to emphasize the importance of a skincare routine that includes utilizing a cleanser and moisturizer. Helping males understand the existing research indicating that face washing can help males with acne, microbiome, razor burn, and folliculitis could potentially increase their willingness to have a consistent face washing and moisturizing routine. 

Although the difference between genders in our study was not statistically significant (p=0.098), it indicates that there was uncertainty on how often participants should wash their faces each day. While many studies and the AAD agree that washing your face twice a day per day is beneficial, it is important that patients discuss this with their healthcare provider for more specific guidance. In our study, female-identifying participants are more likely to receive their skincare information from a healthcare professional (p<0.001). It is important for non-female identifying individuals to also receive their information from a healthcare professional since each person’s needs are unique. 

This study had a few limitations. One significant limitation was that only 124 responses were analyzed, which decreased the generalizability of the study. Additionally, there were 33 male identifying participants which limited proper representation. Despite the majority of the population being female-identifying, the results were still statistically significant. While the audience was the general population, 95% of the respondents had a Bachelor’s degree or higher. This made it difficult to assess if education was a factor in a respondent’s facial cleansing routine. Moreover, this may have skewed the results to show a consistent skincare routine. 

Statistically significant results were seen for female-identifying participants receiving their skincare information from a healthcare professional and/or healthcare professional's social media. This study showed that there needs to be more research and outreach for male-identifying participants. Future research should aim to redistribute the survey with a focus on expanding the sample size to increase generalizability. Additionally, efforts should be made to increase the sample size by targeting more male-identifying participants. Furthermore, the survey should be distributed to include participants with broader education levels by targeting subjects with an education level below a Bachelor’s degree. According to the U.S. Census Bureau, in 2021, the highest level of education for Americans above the age of 25 was a Bachelor's degree (23.5%) and an advanced degree such as a Master’s or Doctorate (14.4%) [15]. Therefore, the results of this survey did not represent the majority of the general population. 

## Conclusions

There is a strong correlation between identifying females and following AAD facial skincare recommendations. Female identifying participants are motivated by hygiene, beauty/anti-aging, and health (acne, psoriasis, etc.). Female-identifying participants are more likely to seek skincare routine information from a healthcare professional. There is uncertainty among the participants on how often they should wash their face each day. Designing infographic pamphlets with face cleansing recommendations for distribution at medical offices, particularly primary care offices, can help further educate the general public on face washing, as well as incorporating skincare hygiene into routine medical visits such as annual physicals. Additionally, more effort should be made to educate men on the benefits of a good skincare routine. The survey should be repeated with the aim of larger sample size and more male-identifying participants. 

## Appendices

Appendix A 

**Table 3:** Skincare routine survey questions.

Survey Questions				
Question number	Question text	Question type	Response Options	Notes/Instructions
Q1	Online survey (Alternate consent)	Complete checkbox	18 and older to participate -voluntary consent to participate in a survey	N/A
Q2	What is your age?	Multiple choice	18-29, 30-49, 50-69, 70-79, 80-89	Select one option
Q3	What gender do you identify as?	Multiple choice	Male, female, transgender male, transgender female, non-binary, other, prefer not to answe.r	Select one option
Q4	What race do you identify as?	Multiple choice	Caucasian, African American, American Indian or Alaskan Native, Asian, Native Hawaiian or Pacific Islander, Hispanic, mixed race	Select one option
Q5	What is the highest level of education you completed?	Multiple choice	High school/GED, Associate’s, Bachelors, Master’s, Professional, Doctorate, Professional certificate	Select one option
Q6	What is your marital status?	Multiple choice	Single (never married), long-term committed relationship, living with partner, married, separated, widowed, divorced	Select one option
Q7	What is your skin type?	Multiple choice	Normal, dry, oily, combination (oily and dry), I am not sure	Select one option
Q8	Do you have a daily skincare routine?	Yes/No	Yes, No	Select one option
Q9	How often do you wash your face?	Multiple choice	Once per day, twice per day, more than twice per day, a few times per week, I don’t wash my face regularly.	Select one option
Q10	What do you use to wash your face?	Multiple choice	Body wash (including 2-in-1 products), shampoo, bar/hand soap, facial cleanser, water, I don’t wash my face.	Select one option
Q11	Do you use an alcohol-free cleanser?	Multiple choice	Yes, No, I don’t know, I don’t use a facial cleanser	Select one option
Q12	What do you use to apply the cleanser?	Multiple choice	Washcloth, mesh sponge, fingertips, I don’t use a facial cleanser	Select one option
Q13	What is the temperature when you are washing your face?	Multiple choice	Cold, warm, hot, I do not wash my face	Select one option
Q14	Do you apply a facial moisturizer after using a facial cleanser?	Yes/No	Yes, No	Select one option
Q15	What additional skincare products do you use?	Multiple choice	Sunscreen, toner, serums, vitamin C products, retinol/retinoid, glycolic acid, niacinamide products, N/A	Select all that apply
Q16	Do you use a facial cleanser after heavy sweating or a workout?	Yes/No	Yes, No	Select one option
Q17	What motivates you to have a skincare routine?	Multiple choice	Hygiene, Health (ex.acne, psoriasis, eczema, etc.), beauty/anti-aging, confidence, influence from significant other, I don’t have a skincare routine, other.	Select all that apply if choosing “other” space to type
Q18	Where do you receive your skincare routine information?	Multiple choice	Google/internet, social media influencers (Tiktok, Instagram, Facebook, etc.), healthcare professionals, healthcare professional’s social media, family/friends/significant other, I don’t have a skincare routine.	Select all that apply
Q19	How did COVID-19 impact your skincare routine?	Multiple choice	My skincare routine improved, my skincare routine worsened, my skincare routine did not change.	Select one option
